# Development of quantitation method for glycated aminophospholipids at the molecular species level in powdered milk and powdered buttermilk

**DOI:** 10.1038/s41598-018-27010-2

**Published:** 2018-06-07

**Authors:** Ai Kodate, Yurika Otoki, Naoki Shimizu, Junya Ito, Shunji Kato, Naoki Umetsu, Teruo Miyazawa, Kiyotaka Nakagawa

**Affiliations:** 1 Food and Biodynamic Chemistry Laboratory, Graduate School of Agricultural Science, Tohoku University, Sendai, 980-0845 Japan; 2 New Industry Creation Hatchery Center (NICHe), Tohoku University, Sendai, 980-0845 Japan; 3 Food and Health Science Research Unit, Graduate School of Agricultural Science, Tohoku University, Sendai, 980-0845 Japan

## Abstract

The Maillard reaction is a nonenzymatic glycation reaction between a reducing sugar and a free amino group, known to naturally occur during heat processing of food. In this study, we especially focused on phosphatidylethanolamine (PE)-linked Amadori products (Amadori-PE) in powdered milk, since the analysis of these products at the molecular species level has not yet been evaluated. Analysis of Amadori-PE was conducted by using liquid chromatography-tandem mass spectrometry in three different modes. The main Amadori-PE species in a powdered milk sample were first identified as 34:1, 36:1, 36:2 and 36:3 in the total ion current mode. Additionally, by using the characteristic product ions observed in the presence of sodium, we quantified the main Amadori-PE species in the multiple reaction monitoring mode, and evaluated their total concentrations in the precursor ion scan (PIS) mode for the first time. Powdered milk contained much Amadori-PE with concentrations ranging from 4.3 to 8239 mg/100 g, quantified by the PIS mode. The newly developed methods represent powerful tools for detailed analysis of glycated lipids including Amadori-PE in powdered milk, which may further be applied to research relating to infant food and nutrition.

## Introduction

**Figure 1:**
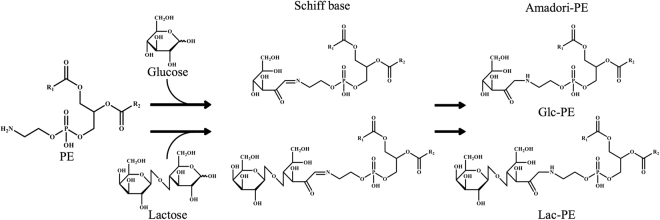
Scheme for the glycation of PE. Reacting glucose or lactose with the amino group of PE yields the stable PE-linked Amadori product (Amadori-PE) via an unstable Schiff base. Glc-PE, Amadori product derived from glucose and PE; Lac-PE, Amadori product derived from lactose and PE.

The Maillard reaction is a nonenzymatic glycation reaction between a reducing sugar and a free amino group, known to naturally occur during heat processing of food, producing the various colours and flavours of processed food. The Maillard reaction is typically considered as the glycation of the free amino group in amino acids or proteins, but it has become known that the free amino groups of aminophospholipids are also targets for the glycation, which expanded the concept of the Maillard reaction^1–4^. For example, a reaction between phosphatidylethanolamine (PE) and sugar yields PE-linked Amadori products (Amadori-PE; Fig. 1) via an unstable Schiff base as its intermediate^5–10^. Amadori-PE glycated with glucose (Glc-PE) and with lactose (Lac-PE) are found in various processed foods, especially powdered milk (powdered bovine milk) contains a significant amount of Amadori-PE with concentrations ranging from 3.2 to 11.2 mg/100 g, corresponding to about 30% of total PE^11,12^. This is presumably produced from the monosaccharides and PE in the ingredients of powdered milk being heated during the manufacturing process. Relatively large amounts of glycated PE is considered to affect the nutritional value and physical properties of powdered milk.

The non-glycated (native) PE in powdered milk exists as several molecular species including 1-palmitoyl-2-oleoyl-*sn*-glycero-3-phosphoethanolamine (34:1 PE), 1-stearoyl-2-oleoyl-*sn*-glycero-3-phosphoethanolamine (36:1 PE), 1-stearoyl-2-linoleoyl-*sn*-glycero-3-phosphoethanolamine (36:2 PE), 1,2-dioleoyl-*sn*-glycero-3-phosphoethanolamine (36:2 PE) and 1-oleoyl-2-linoleoyl-*sn*-glycero-3-phosphoethanolamine (36:3 PE) as a component of the milk fat globule membrane^12^. As these species are considered to be beneficial sources of fatty acids (*e.g*. linoleic acid) that act as a lipid mediator in immunological protection^13,14^, investigating the molecular species of PE is essential. Considering that Amadori-PE is readily formed in powdered milk, it is necessary to identify the species and amounts of PE glycated with glucose or with lactose. However, the analysis of Amadori-PE (Glc-PE and Lac-PE) in powdered milk at the molecular species level has not yet been evaluated.

In the present study, we analysed Glc-PE and Lac-PE molecular species in powdered milk by using liquid chromatography-tandem mass spectrometry (LC-MS/MS) in three different modes. With the use of the total ion current (TIC) mode, the main Glc-PE and Lac-PE species in powdered milk were identified. To developing the specific quantification method, characteristic product ions of each Glc-PE and Lac-PE species were found by MS/MS analysis in the presence of sodium ion. By using the characteristic product ions, we successfully achieved to quantify the main Glc-PE and Lac-PE species in the multiple reaction monitoring (MRM) mode. Then we attempted to estimate the total Glc-PE and Lac-PE concentration in the precursor ion scan (PIS) mode.

## Methods

### Materials

1-Palmitoyl-2-oleoyl-*sn*-glycero-3-phosphoethanolamine (34:1 PE), 1-stearoyl-2-oleoyl-*sn*-glycero-3-phosphoethanolamine (36:1 PE) and 1-stearoyl-2-linoleoyl-*sn*-glycero-3-phosphoethanolamine (36:2 PE) were purchased from Avanti Polar Lipids (Alabaster, AL, USA). D-Glucose was obtained from Wako (Osaka, Japan). β-Lactose was obtained from Sigma-Aldrich (St Louis, MO, USA). All organic solvents were HPLC grade (Wako, Osaka, Japan) and other regent purities were more than 99.5%.

### Phospholipid extraction from powdered milk

A powdered milk sample derived from buttermilk (milk phospholipid concentrate, MPLC) was kindly provided from MEGMILK SNOW BRAND (Tokyo, Japan). MPLC was produced by following procedures: The raw buttermilk sterilized at 100–140 °C for a few seconds was concentrated by evaporation under heat, reduced pressure, and spray drying. The resultant powdered buttermilk was dissolved in water, and proteins were removed from the liquid under low pH. The powdered buttermilk was then concentrated and powderized by using ultrafiltration and freeze drying, respectively.

For LC-MS/MS analysis in the TIC mode, the total lipid of MPLC was extracted by Folch’s method^15^. In brief, 5 g of MPLC was mixed with 9 mL of 0.9% NaCl (w/v) aqueous solution (containing 1 mM ethylenediaminetetraacetic acid). Then the solution was further mixed with 36 mL of chloroform: methanol (2:1, v/v) containing 0.002% butylated hydroxytoluene, and subjected to centrifugation at 1,000 × *g* for 20 min at 4 °C. The resultant chloroform layer (under organic layer) was collected and the remaining aqueous layer (methanol-water layer) was mixed with Folch’s theoretical under phase. The under chloroform layers were combined, evaporated and dried under nitrogen gas. The dried extract was reconstituted in 2 mL of chloroform:isopropanol (2:1, v/v), and 2 mL of this mixture was subsequently loaded onto a silica Sep-Pak cartridge (10 g, Waters, Tokyo, Japan) equilibrated with chloroform:isopropanol (2:1, v/v). The phospholipid fraction including Amadori PE was eluted with 10 mL of methanol. The solvent was evaporated under nitrogen gas, and then reconstituted in 100 mL of methanol. The resultant phospholipid fraction was diluted 1,000-fold and 1 µL was analysed with LC-MS/MS in the TIC mode.

For LC-MS/MS analysis in the MRM and PIS modes, MPLC and other powdered milk samples (sample A, B and C) purchased from a local supermarket in Sendai, Japan were extracted by methanol precipitate^16^, as follows. Eighty microliters of powdered milk dissolved in water (20 mg/mL) was mixed with 20 µL of 25 µM aqueous ethylenediaminetetraacetic acid and 500 µL of methanol containing 0.002% butylated hydroxytoluene. The mixture was centrifuged at 1000 × *g* for 5 min at 4 °C, and the resultant supernatant was collected. Five hundred microliters of methanol was added to the precipitate, which was then mixed and centrifuged at 1000 × *g* for 15 min at 4 °C. The supernatants were collected and combined. Five hundred microliters of the sample solution was subjected to a silica Sep-Pak cartridge (100 mg, Waters, Tokyo, Japan) in order to remove polar impurities. Subsequently, 1.5 mL of methanol: water (9:1, v/v) was loaded to elute Amadori-PE. Under the optimized LC-MS/MS conditions, 10 μL of the powdered milk extract was injected into the LC-MS/MS system in the MRM or PIS mode.

### Extraction method validation

The recovery rates, accuracy and precision were evaluated for extraction method validation. MPLC and sample A spiked or non-spiked Lac-PE standards (440 pmol of Lac-PE, 36:1 Lac-PE and 36:2 Lac-PE) were extracted by methanol precipitation mentioned above. Lac-PE, 36:1 Lac-PE and 36:2 Lac-PE were quantitated by LC-MS/MS operated in MRM and the concentrations were calculated based on each external standard curve. Recovery rates were determined by comparing concentration obtained from MPLC or sample A spiked standard solution with each blank. Accuracy was expressed as the percentage different between expected and observed concentration. Precision was provided by the coefficient of variation from blank sample concentration. The acceptance criterion for accuracy and precision was 25%. Measurements were carried out in 3 replicate.

### TIC analysis of Amadori-PE species in powdered milk

A Shimadzu liquid chromatography system consisting of a vacuum degasser, a quaternary pump, and an auto sampler (Shimadzu, Kyoto, Japan) was equipped with a 4000 QTRAP mass spectrometer (SCIEX, Tokyo, Japan). Glc-PE and Lac-PE were analysed by using a silica column (Inertsil SIL-100A, 3 µm, 2.1 × 100 mm, GL Science, Tokyo, Japan) with a binary gradient consisting of solvent A (chloroform-methanol-28% ammonia solution 80:19.5:0.5 (v/v/v)) and solvent B (chloroform-methanol-water-28% ammonia solution 60:34:5.5:0.5 (v/v/v/v)). The gradient profile was as follows: 0–20 min, 0–100% B linear; 20–30 min, 100% B; 30–40 min, 0% B. The flow rate was 0.2 mL/min, and the column temperature was maintained at 40 °C. Positive electrospray ionization (ESI) was used with the following experimental parameters: curtain gas, 20 psi; ion spray voltage, 5500 V; temperature, 500 °C; ion source gas 1, 60 psi; ion source gas 2, 70 psi; declustering potential, 120 V; entrance potential, 10 V. Positive ion spectra were collected in the *m/z* range of 700–1100.

### MS/MS fragmentation analysis of Amadori-PE standards

Based on the aforementioned TIC study results, Glc-PE and Lac-PE standards were synthesised by reacting commercially available native PE (34:1, 36:1 and 36:2) with either glucose or lactose, as previously described^15^.

To evaluate MS/MS fragmentation patterns, the synthesised Glc-PE and Lac-PE standards (1 nmol) were each dissolved in 1 mL of methanol containing either 5 mM ammonium acetate or 0.1 mM sodium acetate, and directly infused (2.5 µL/min) into a quadrupole-time-of-flight mass spectrometer (micrOTOF-QII, Bruker Daltonics, MA, USA) under positive ESI. The parameters were as follows: capillary, 6000 V; collision energy, 10.0 eV; N_2_ nebulizer gas pressure, 1.6 bar; N_2_ dry gas flow rate, 8.0 L/min; N_2_ dry gas temperature, 180 °C. The collision cell parameters were as follows: collision RF, 1400 Vpp; transfer time, 120 µs; pre-pulse storage; 15 µs. Positive ion spectra were collected in the *m/z* range of 200–1200.

### MRM analysis of main Amadori-PE species

To quantify Glc-PE and Lac-PE at the molecular species level, LC-MS/MS analysis in the MRM mode was conducted with a 4000 QTRAP mass spectrometer. An ODS column (Atlantis T3, 2.1 × 100 mm; Waters, Tokyo, Japan) was used with a binary gradient consisting of solvent A (water containing 0.1 mM sodium acetate) and solvent B (methanol containing 0.1 mM sodium acetate). The gradient profile was as follows: 0–5 min, 65% B; 5–25 min, 65–100% B linear; 25–40 min, 100% B; 40–40.1 min, 100–65% B linear; 40.1–45 min, 65% B. The flow rate was as follows; 0–5.0 min, 0.25 mL/min; 5.1–40.0 min, 0.2 mL/min; 40.1–45 min, 0.25 mL/min, and the column temperature was maintained at 40 °C. MS/MS parameters of Glc-PE and Lac-PE were optimized with their standards under ESI with the following experimental parameters: curtain gas, 20 psi; collision gas, 8 psi; ion spray voltage, 5500 V; temperature, 500 °C; ion source gas 1, 70 psi; ion source gas 2, 80 psi. Other parameters and MRM pairs are described in Table 1. The concentrations of 34:1 Glc-PE, 36:1 Glc-PE, 36:2 Glc-PE, 34:1 Lac-PE, 36:1 Lac-PE and 36:2 Lac-PE in powdered milk were calculated from their respective standard curves. To evaluate recovery rates, analysis was performed on MPLC phospholipid fractions, which were either non-spiked or spiked with 50 pmol of 6 Amadori-PE standards before extraction. Recovery rates were determined by comparing the concentration of Amadori-PE in non-spiked MPLC with MPLC spiked with Amadori-PE standards. The matrix factor was determined by comparing the response of Amadori-PE in the presence of the matrix (*e.g*. MPLC phospholipids) with that in the absence of the matrix^17^. Additionally, native PE was analysed in the MRM mode as previously described^18^. For MRM and PIS calibration curves, 0.5–10 pmol and 1–20 pmol of each standard was analyzed by triplicate, respectively.

**Table 1:** Optimal MS/MS parameters for the detection of Glc-PE and Lac-PE in the MRM and PIS modes.

Mode	Amadori-PE	Species	Precursor ion (*m/z*)	Product ion (*m/z*)	DP (V)	EP (V)	CE (V)	CXP (V)
MRM	Glc-PE	34:1	902.6	326.1	120	10	45	18
		36:1	930.6	326.1	102	10	53	18
		36:2	928.6	326.1	102	10	53	18
	Lac-PE	34:1	1064.6	488.1	125	10	56	30
		36:1	1092.7	488.1	115	10	57	28
		36:2	1090.6	488.1	115	10	57	28
PIS	Glc-PE			326.1	90–156	10	45	18
	Lac-PE			488.1	115–166	10	56	28

### PIS analysis of total Amadori-PE

To evaluate total amounts of Glc-PE and Lac-PE, LC-MS/MS analysis in the PIS mode was conducted. ESI MS/MS parameters of Glc-PE and Lac-PE were optimized with their standards under the following parameters: curtain gas, 20 psi; collision gas, 4 psi; ion spray voltage, 5500 V; temperature, 500 °C; ion source gas 1, 15 psi; ion source gas 2, 30 psi. Positive ion spectra were collected in the *m/z* range of 1000–1200. Other analytical conditions and fixed ions of PIS are described in Table 1.

## Results and Discussion

### TIC analysis of Amadori-PE species in powdered milk

**Figure 2:**
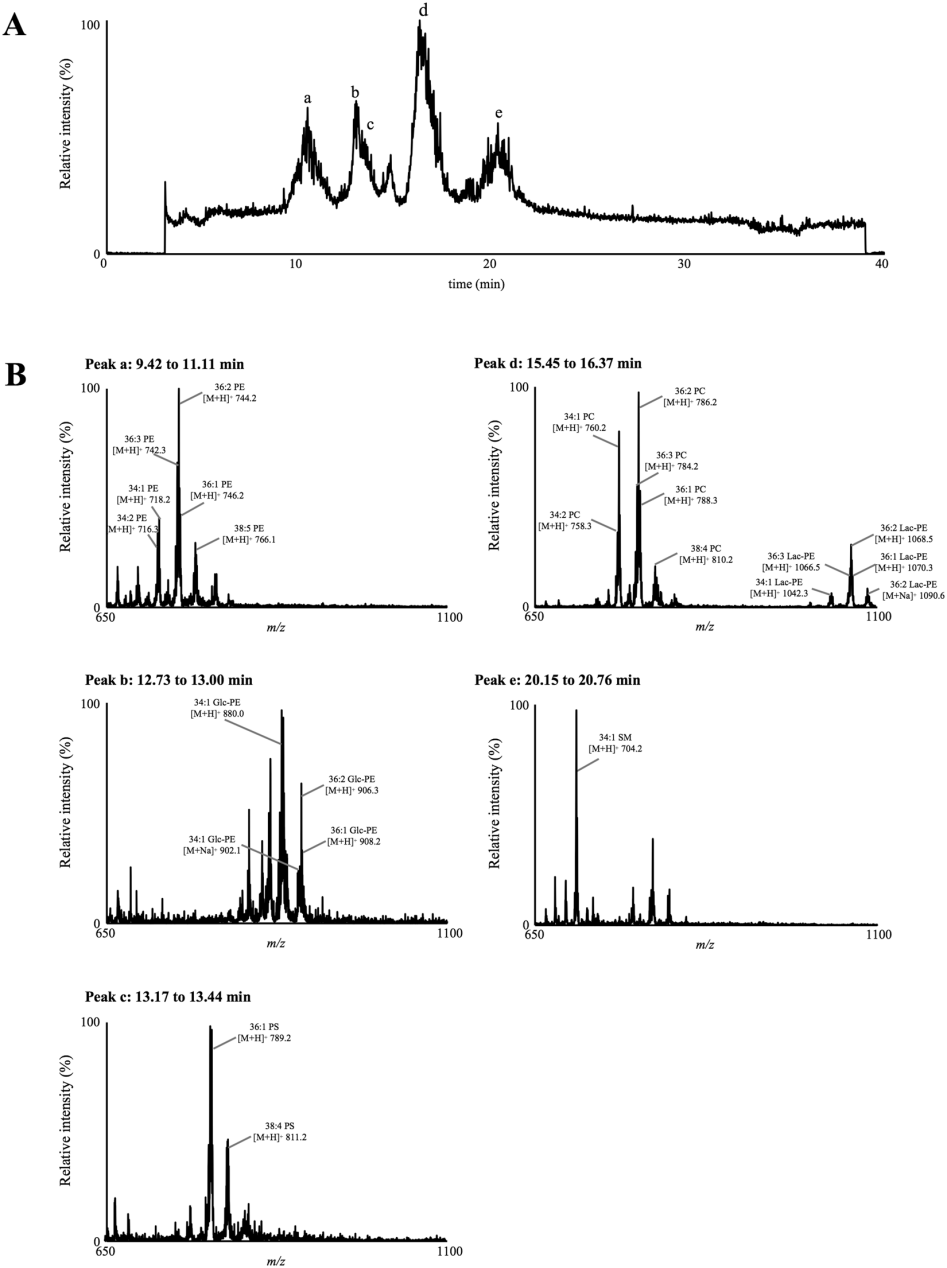
TIC chromatogram of phospholipid classes in MPLC (**A**). Q1 mass spectra of peaks a (9.43–11.11 min), b (12.73–13.00 min), c (13.17–13.44 min), d (15.45–16.37 min) and e (20.15–20.76 min) (**B**). According to the mass spectra, each peak (a–d) was identified as PE, Glc-PE, phosphatidylserine (PS), phosphatidylcholine (PC) & Lac-PE, and sphingomyelin (SM), respectively. Detailed analytical conditions are described in the Methods section.

As MPLC is produced from buttermilk powder, lipids including PE are rich. Hence, we assumed that it contained large amounts of Amadori-PE species, and would be an effective powdered milk sample for the identification of Glc-PE and Lac-PE species. To simultaneously evaluate Amadori-PE and phospholipid classes in MPLC, the prepared phospholipid fraction was analysed with normal phase in the TIC mode^19,20^. As shown in Fig. 2A, 5 main peaks were detected, and the identity of each peak was determined from the mass spectrum as follows: PE (a), Glc-PE (b), phosphatidylserine (PS) (c), phosphatidylcholine (PC) and Lac-PE (d) and sphingomyelin (SM) (e) (Fig. 2B). As expected, TIC analysis confirmed that MPLC contained large amounts of Amadori-PE, especially Lac-PE. The main phospholipid species were PE, PC, SM and PS in MPLC as comparable with previous reports^21,22^. Although PS is another phospholipid that contains an amino group that could react with reducing sugars, glycated PS was not detected in MPLC as previously reported^11^, possibly due to the low content of PS. Glycation end products such as carboxymethyl-PE, N-carboxyacyl-PE and carboxyethylphosphatidyl-PE were also not observed in the Q1 mass spectrum. Thus, it was suggested that the main glycation product in powdered milk is Amadori-PE, as previously reported^9,23,24^.

Based on the mass spectrum of Glc-PE (Fig. 2B), the main molecular species of Glc-PE present in MPLC were identified as 34:1, 36:1, 36:2 and 36:3; the *sn*-1/*sn*-2 fatty acids components were predicted to be 16:0 (palmitoyl)/18:1 (oleoyl), 18:0 (stearoyl)/18:1, 18:0/18:2 (linoleoyl) or 18:1/18:1, 18:1/18:2, respectively. The main molecular species of Lac-PE were the same as that of Glc-PE (Fig. 2B). To the best of our knowledge, this is the first study identifying the molecular species of Amadori-PE in powdered milk. The fact that the main Glc-PE and Lac-PE species were similar with the main native PE species in MPLC indicates that the glycation of PE depends on the abundance of the native PE species and that glycation occurs during the manufacturing process.

In terms of the mass spectra, Glc-PE and Lac-PE in MPLC were detected in the form of both [M + H]^+^ and [M + Na]^+^ (Fig. 2B). The formation of [M + Na]^+^ may be attributed to the presence of sodium in MPLC and/or analytical instruments, and suggest that Amadori-PE easily forms sodium adducts. The abundance ratio of [M + H]^+^ and [M + Na]^+^ fluctuated even within the same analysis, thus we presumed that analysis using [M + H]^+^ may cause unstable quantification.

### MS/MS analysis of Amadori-PE standards

Based on the results from TIC studies (Fig. 2), we selected 3 Glc-PE species (34:1, 36:1, 36:2) and 3 Lac-PE species (34:1, 36:1, 36:2) as targets of quantification, and each standard was synthesised from commercially available native PE. In establishing quantification methods with LC-MS/MS, identification of characteristic product ions is important to provide high selectivity. We have previously found that characteristic product ions were generated under the presence of sodium in other phospholipids^25,26^, and thereby to explore useful product ions for the quantification of Amadori-PE, the synthesised 6 standards (34:1 Glc-PE, 36:1 Glc-PE, 36:2 Glc-PE, 34:1 Lac-PE, 36:1 Lac-PE and 36:2 Lac-PE) were analysed in the presence or absence of sodium.

**Figure 3:**
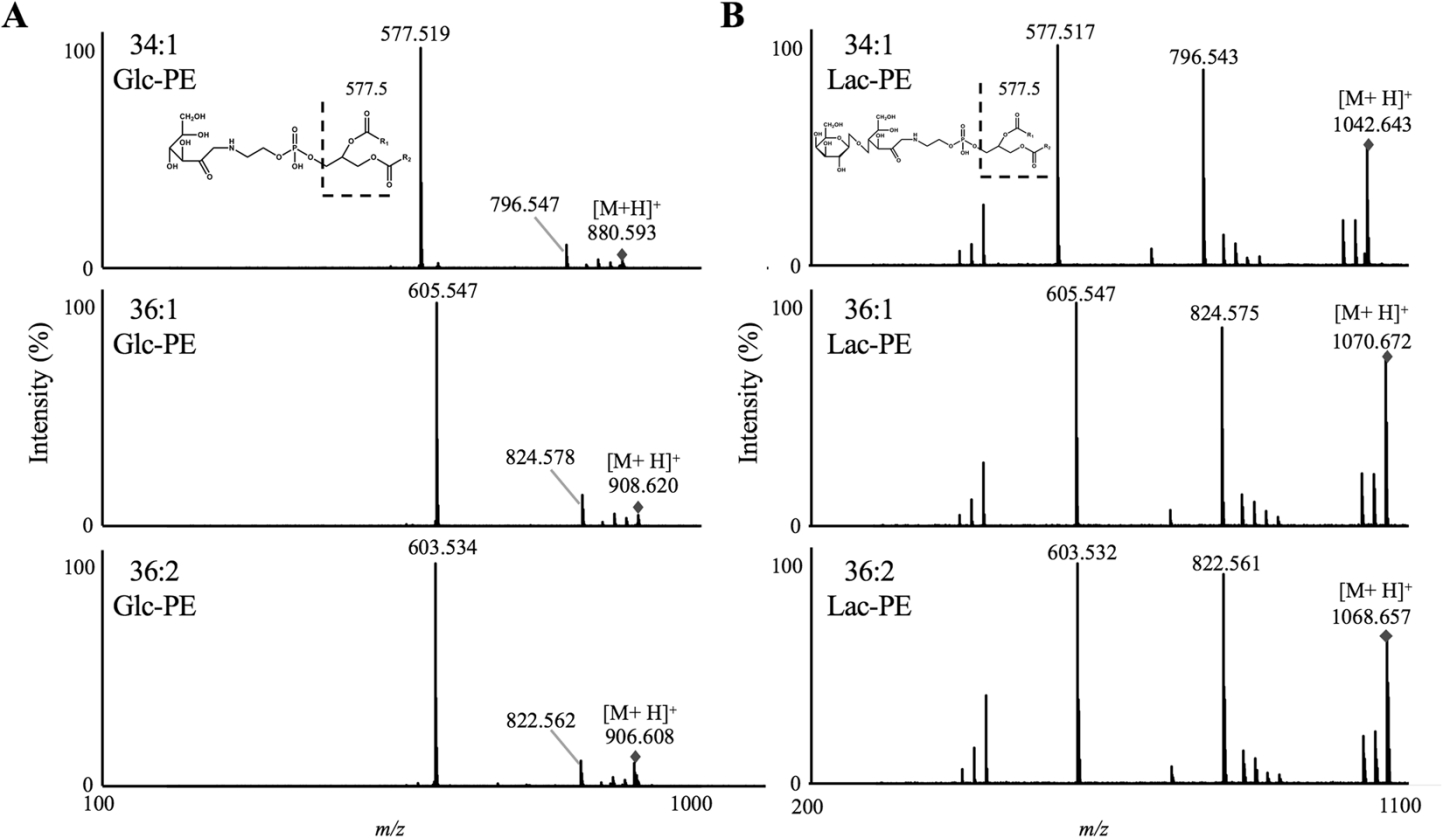
Product ion mass spectrum of 34:1, 36:1 and 36:2 Glc-PE (**A**) and 34:1, 36:1 and 36:2 Lac-PE (**B**). Each standard (1 µM) was infused directly into the MS/MS (2.5 µL/min) in the absence of sodium. Detailed analytical conditions are described in the Methods section.

In the absence of sodium, collision-induced dissociation (CID) of [M + H]^+^ produced each of the following product ions: 34:1 Glc-PE (*m/z* 577.519) and Lac-PE (*m/z* 577.517), 36:1 Glc-PE (*m/z* 605.547) and Lac-PE (*m/z* 605.547) and 36:2 Glc-PE (*m/z* 603.534) and Lac-PE (*m/z* 603.532) (Fig. 3). These product ions were derived from the neutral loss of the glycated polar head, and contained *sn*-1 and *sn*-2 fatty acids of each Amadori-PE as previously reported^27^. However, these product ions were also generated from native PE corresponding to the loss of a phosphate group, and therefore were less specific to Amadori-PE.

**Figure 4:**
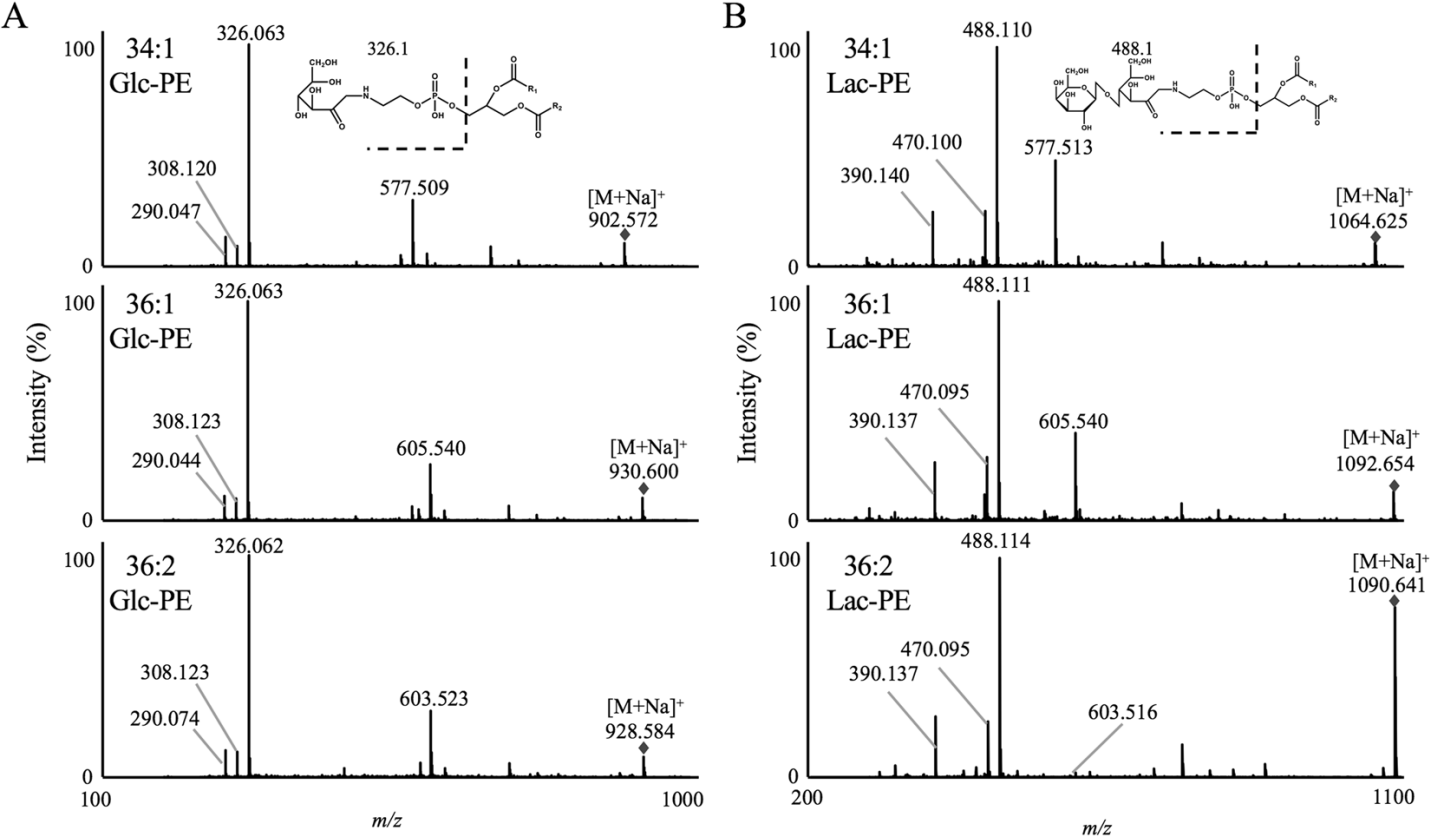
Product ion mass spectrum of 34:1, 36:1 and 36:2 Glc-PE (**A**) and 34:1, 36:1 and 36:2 Lac-PE (**B**). Each standard (1 µM) was infused directly into the MS/MS (2.5 µL/min) in the presence of sodium. Detailed analytical conditions are described in the Methods section.

Conversely, in the presence of sodium, CID of [M + Na]^+^ showed each of the following product ions: 34:1 Glc-PE [M + Na]^+^ yielded *m/z* 577.509 as in [M + H]^+^, though, *m/z* 326.063, 308.120 and 290.047 were characteristically detected in the presence of sodium (Fig. 4). According to each mass spectrum and predicted fragmentation pattern, *m/z* 326.063 was assumed as [H_3_PO_4_CH_2_CH_2_NHC_6_H_10_O_5_Na]^+^ (exact mass: 326.0617). Additionally, *m/z* 308.120 and 290.047 corresponded to a neutral loss of either one or two molecules of H_2_O from *m/z* 326.063. These fragmentation patterns were observed from 36:1 Glc-PE and 36:2 Glc-PE [M + Na]^+^ as well. Similarly, 34:1 Lac-PE [M + Na]^+^ predominantly yielded *m/z* 488.110, corresponding to [H_3_PO_4_CH_2_CH_2_NHC_12_H_20_O_10_Na]^+^ (exact mass 488.1145) (Fig. 4**)**. The dehydration product of *m/z* 488.110, *m/z* 470.100, was also detected. To the best of our knowledge, [H_3_PO_4_CH_2_CH_2_NHC_6_H_10_O_5_Na]^+^ and [H_3_PO_4_CH_2_CH_2_NHC_12_H_20_O_10_Na]^+^ were detected for the first time. The product ions were derived from the neutral loss of *sn*-1 and *sn*-2 fatty acids along with the glycerol backbone, and contained the glycated polar head of each Amadori-PE. Contrary to the product ions produced in the absence of sodium (*e.g. m/z* 577.519), the product ions produced in the presence of sodium were not generated from native PE, and were specifically diagnostic for Glc-PE and Lac-PE. Thus, we presumed that the use of these product ions as unilateral pairs of MRM analysis would enable quantification with higher selectivity. In addition, these product ions were mutual to all Glc-PE or Lac-PE regardless of the difference in molecular species, and were considered effective to be used in the PIS analysis to evaluate total Glc-PE or Lac-PE.

Regarding the adduct ions, only [M + Na]^+^ was detected as the precursor ion in the presence of sodium (Fig. 4), resolving the aforementioned fluctuation problem in the abundance ratio of [M + H]^+^ and [M + Na]^+^, thereby ensuring quantitativeness within analysis. This was another advantage in analysing Amadori-PE in the presence of sodium as opposed to the absence of sodium.

### MRM and PIS analysis of Amadori-PE in powdered milk

#### MRM analysis of Amadori-PE

**Figure 5:**
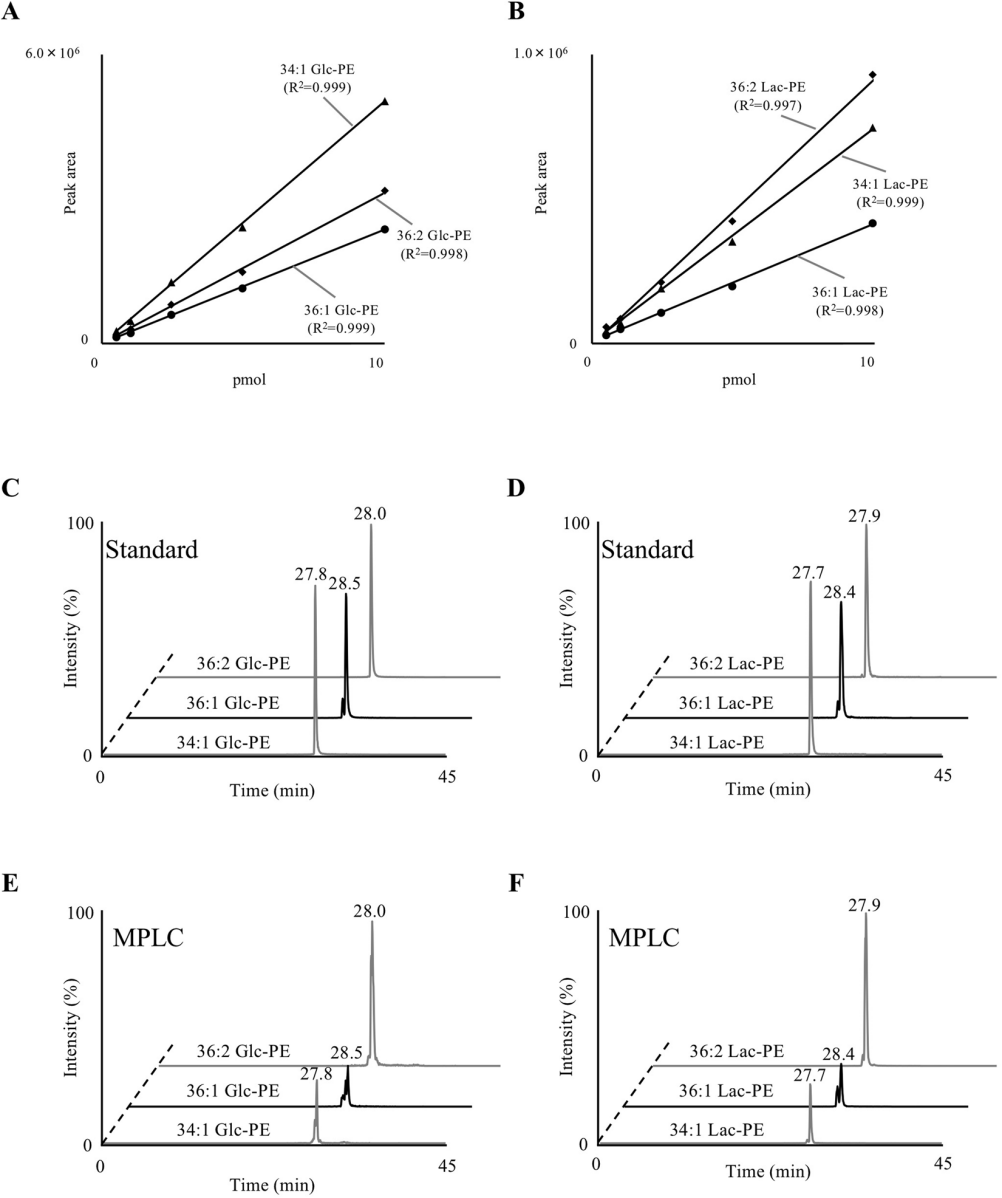
MRM analysis of Glc-PE (**A**,**C**,**E**) and Lac-PE (**B**,**D**,**F**). Calibration curves of 34:1, 36:1 and 36:2 Glc-PE (**A**) and 34:1, 36:1 and 36:2 Lac-PE (**B**). Different amounts of Glc-PE and Lac-PE (0.5–10 pmol) were analysed in the MRM mode. MRM chromatograms of Glc-PE (**A**,**C**) and Lac-PE (**B**,**D**) in the presence of sodium. A mixture of standards (10 pmol each) (**C**,**D**) and MPLC (**E**,**F**) were analysed by LC-MS/MS. Detailed analytical conditions are described in the Methods section.

With the use of the characteristic product ions of Glc-PE (*e.g. m/z* 326.063) and Lac-PE (*e.g. m/z* 488.114) that were identified by MS/MS analysis in the presence of sodium, MRM analysis was conducted for the quantification of Amadori-PE in MPLC at the molecular species level. MRM is frequently used for quantification of Amadori-PE^18,27^ as MRM enables selective and sensitive detection of target compounds^28,29^. In the present study, since the characteristic product ions of Glc-PE and Lac-PE were found (Fig. 4), we used these product ions as unilateral pairs and determined the MRM pairs of six different Amadori-PE species (34:1 Glc-PE: 902.6/326.1, 36:1 Glc-PE: 930.6/326.1, 36:2 Glc-PE: 928.6/326.1, 34:1 Lac-PE: 1064.6/488.1, 36:1 Lac-PE: 1092.7/488.1 and 36:2 Lac-PE: 1090.6/488.1). For the separation of the molecular species of Amadori-PE, a reverse phase column (ODS) was used since it had generally been adopted to separate molecular species in phospholipid analysis^30^. A gradient flow of methanol and water both containing 0.1 mM sodium acetate was used as the mobile phase. In general, nonvolatile salts, including sodium, are considered to adversely affect MS instruments^31^. However, in the developed method, the concentration of sodium acetate was set at 0.1 mM, a low concentration at which stable analysis was achieved in previous reports^16,25,26^. The external calibration curves were found to exhibit good linearity within the range of 0.5 to 10 pmol per injection (Fig. 5A,B). Under the optimized conditions, each Amadori-PE standard was detected as a clear single peak (Fig. 5C,D). The detection limits of the main Glc-PE and Lac-PE species were 50 fmol, superior to the previous reports^18,27^.

For the precise quantification of the main Amadori-PE species in MPLC, the extraction method was investigated as to whether it could properly extract each Amadori-PE species in MPLC. The recovery rates of Lac-PE species were 73.0–87.8% (accuracy:12.2–27.0, precision: 7.5–20.3) and 101.8–108.0% (accuracy:1.8-8.0, precision: 3.5–4.3) in MPLC and powdered milk sample A, respectively. The acceptance criterion for accuracy and precision of 25% was almost achieved for extraction of the main Amadori-PE species. In addition, we evaluated ion suppression as it may affect quantification due to the large content of phospholipids relative to Amadori-PE in powdered milk, and the difficulty of their chromatographic separation. The matrix factors were 97.1–98.5% for Glc-PE and 97.4–98.8% for Lac-PE, thus we confirmed that the matrix effects were considered negligible.

As evaluation of the LC separation and extraction method was achieved, Glc-PE and Lac-PE in MPLC were analysed in the MRM mode. Glc-PE and Lac-PE in MPLC were detected as clear single peaks (Fig. 5E,F). By using external calibration curves, concentrations of the molecular species of Glc-PE and Lac-PE in MPLC were quantified as 19.5 mg/100 g for 34:1 Glc-PE, 23.6 mg/100 g for 36:1 Glc-PE, 100.0 mg/100 g for 36:2 Glc-PE, 115.1 mg/100 g for 34:1 Lac-PE, 1820.4 mg/100 g for 36:1 Lac-PE, and 4837.9 mg/100 g for 36:2 Lac-PE. The abundance of the molecular species of Glc-PE and Lac-PE in MPLC was 36:2, 36:1, and 34:1, in descending order. In agreement with the TIC analysis (Fig. 2), MPLC contained larger amounts of Lac-PE than Glc-PE. In the same way, other typical powdered milks were analysed in the MRM mode. The concentration of Glc-PE and Lac-PE in these milks are also described in Table 2.

**Table 2:** Concentrations of Glc-PE and Lac-PE in powdered milk obtained with the MRM and PIS modes.

	MRM						PIS	
	Glc-PE			Lac-PE			Total Glc-PE	Total Lac-PE
	34:1	36:1	36:2	34:1	36:1	36:2		
mg/100 g								
MPLC	19.5 ± 3.0	23.6 ± 3.8	100.0 ± 2.8	115.1 ± 3.2	1820.4 ± 31.9	4837.9 ± 367.2	480.3 ± 9.0	8239.6 ± 506.2
A	1.8 ± 0.1	2.5 ± 0.1	2.9 ± 0.1	1.5 ± 0.2	1.6 ± 0.2	7.0 ± 0.4	7.8 ± 0.7	11.6 ± 0.2
B	0.9 ± 0.05	0.8 ± 0.09	1.9 ± 0.1	2.1 ± 0.1	1.7 ± 0.2	6.7 ± 0.3	4.3 ± 0.3	18.7 ± 0.6
C	0.7 ± 0.05	0.4 ± 0.06	1.4 ± 0.1	2.2 ± 0.2	2.1 ± 0.3	9.0 ± 0.4	6.6 ± 0.4	18.9 ± 0.1

Values are described as means ± SDs (n = 3).

### PIS analysis of Glc-PE and Lac-PE

As described above, the utilization of characteristic product ions (Glc-PE: *m/z* 326.1, Lac-PE: *m/z* 488.1) in the MRM analysis enabled accurate quantification of Glc-PE and Lac-PE at the molecular species level. However, quantification of Amadori-PE species with MRM was limited to Amadori-PE species synthesised from commercially available PE. We therefore analysed Amadori-PE in the PIS mode, aiming to evaluate total amounts of Amadori-PE molecular species in MPLC. As the characteristic product ions of Glc-PE and Lac-PE were common within their respective molecular species (Fig. 4), in theory, all Glc-PE and Lac-PE species would be detected in the PIS mode. Hence, in the present PIS analysis, third quadrupole (Q3) is fixed to the fragment masses of analytes (*e.g. m/z* 326.1 and 488.1) for the evaluation of total Glc-PE and Lac-PE.

**Figure 6:**
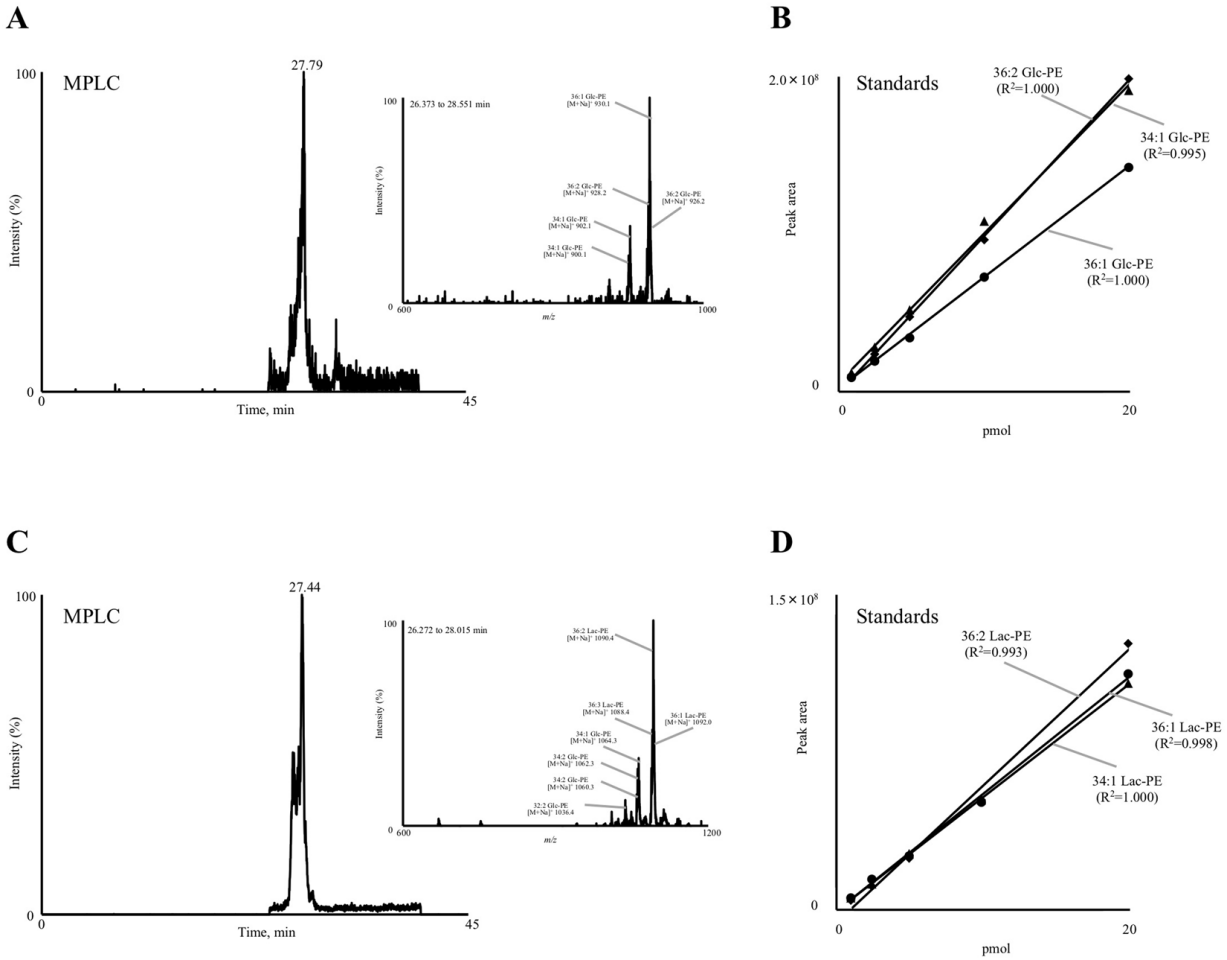
PIS chromatograms of Glc-PE (**A**) and Lac-PE (**C**) in MPLC, analysed in the presence of sodium. Figure inserts show Q1 mass spectra of each peak. Calibration curves of 34:1, 36:1 and 36:2 Glc-PE (**B**) and 34:1, 36:1 and 36:2 Lac-PE (**D**). Different amounts of Glc-PE and Lac-PE (1–20 pmol) were analysed in the PIS mode (PIS of *m/z* 326.1 or 488.1). Detailed analytical procedures are described in the Methods section.

The phospholipid fraction of MPLC was analysed in the PIS mode in the presence of sodium, where Glc-PE and Lac-PE were each detected as single peaks (Fig. 6A,C). From the mass spectrum of each peak, the main species of Glc-PE and Lac-PE found in MPLC were 34:1, 36:1, 36:2 and 36:3 as observed in TIC analysis. With regard to quantification, calibration curves for Glc-PE and Lac-PE were prepared with 36:2 Glc-PE and Lac-PE, respectively, as these were the most abundant species in MPLC. The calibration curves exhibited good linearity within the range of 1 to 20 pmol per injection (Fig. 6B,D). The concentrations of Glc-PE and Lac-PE in MPLC were 480.3 mg/100 g for Glc-PE and 8239.6 mg/100 g for Lac-PE (Table 2). The quantified values in the PIS mode demonstrated higher values than the sum of 34:1, 36:1 and 36:2 calculated from MRM analysis, implying that the evaluation of the total amounts of Amadori PE can be achieved with PIS analysis. Also, the Amadori-PE concentration in MPLC was much higher than that of other powdered milk (Table 2). This may be reflected that MPLC was derived from buttermilk whose phospholipids content was almost 1000 times higher than bovine milk^22,32^.

Importantly, as shown in Fig. 6B and D, the slopes of the individual calibration curves of three Glc-PE or three Lac-PE species slightly varied among species probably due to the difference in ionization efficiency. Therefore, further evaluation (*e.g*. selection of suitable calibration curve) is required to the PIS quantification method in future studies to improve quantitative accuracy.

## Conclusion

In the present study, we aimed to analyse Glc-PE and Lac-PE in powdered milk at the molecular species level by using LC-MS/MS in three different modes. With the TIC mode, the main Glc-PE and Lac-PE species in MPLC were identified as 34:1, 36:1, 36:2 and 36:3. Additionally, by using the characteristic product ions observed in the presence of sodium, we quantified the main Glc-PE and Lac-PE species in the MRM mode, and evaluated their total concentrations in the PIS mode. The methods represent powerful tools to quantify Amadori-PE in powdered milk, which may provide a better understanding of glycated lipids in food and health, especially relating to infant food and nutrition.
